# Timing and Tempo of Early and Successive Adaptive Radiations in Macaronesia

**DOI:** 10.1371/journal.pone.0002139

**Published:** 2008-05-14

**Authors:** Seung-Chul Kim, Michael R. McGowen, Pesach Lubinsky, Janet C. Barber, Mark E. Mort, Arnoldo Santos-Guerra

**Affiliations:** 1 Department of Botany and Plant Sciences, University of California Riverside, Riverside, California, United States of America; 2 Department of Biology, University of California Riverside, Riverside, California, United States of America; 3 Department of Biology, Saint Louis University, St. Louis, Missouri, United States of America; 4 Department of Ecology and Evolutionary Biology and the Natural History Museum and Biodiversity Research Center, University of Kansas, Lawrence, Kansas, United States of America; 5 Jardín de Aclimatación de La Orotava, Tenerife, Canary Islands, Spain; University of California, Berkeley, United States of America

## Abstract

The flora of Macaronesia, which encompasses five Atlantic archipelagos (Azores, Canaries, Madeira, Cape Verde, and Salvage), is exceptionally rich and diverse.

Spectacular radiation of numerous endemic plant groups has made the Macaronesian islands an outstanding area for studies of evolution and speciation. Despite intensive investigation in the last 15 years, absolute age and rate of diversification are poorly known for the flora of Macaronesia. Here we report molecular divergence estimates and rates of diversification for five representative, putative rapid radiations of monophyletic endemic plant lineages across the core eudicot clade of flowering plants. Three discrete windows of colonization during the Miocene and early Pliocene are suggested for these lineages, all of which are inferred to have had a single colonization event followed by rapid radiation. Subsequent inter-archipelago dispersal events into Madeira and the Cape Verdes took place very recently during the late Pliocene and Pleistocene after initial diversification on the Canary Islands. The tempo of adaptive radiations differs among the groups, but is relatively rapid compared to continental and other island radiations. Our results demonstrate that opportunity for island colonization and successful radiation may have been constrained to discrete time periods of profound climatic and geological changes in northern African and the Mediterranean.

## Introduction

**Figure 1:**
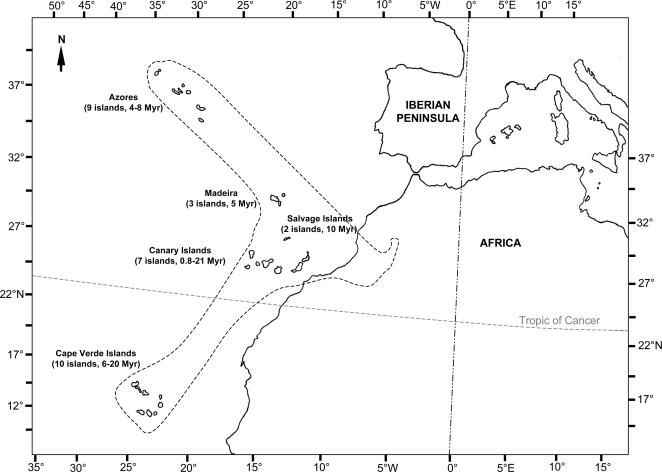
The phytogeographic region of Macaronesia, including five Atlantic volcanic archipelagos (the Azores, the Madeiras, the Salvage Islands, the Canary Islands, and the Cape Verde Islands). The age of current above-sea landmass for each island is from [2].

The phytogeographical region Macaronesia [1] consists of five Atlantic volcanic archipelagos, including the Azores, Madeira, the Salvage Islands, the Canary Islands, and the Cape Verde Islands, as well as a “Macaronesian Enclave” on the African mainland, comprising southern Morocco and the former Spanish West Africa (Fig. 1). The five archipelagos are situated between 15° to 40° N latitude, with distances from the European or African continents varying from 95 to 1600 km. Geological ages of individual islands vary from 0.8 million years (My) for El Hierro to 21 My for Fuerteventura [2], both of which belong to the Canarian archipelago. The influence of moisture-laden northeasterly trade winds combined with altitudes reaching more than 3,700 meters has produced a remarkable diversity of ecological habitats.

The Macaronesian flora displays a number of characteristics typical of oceanic islands, including a high degree of endemism (20% of overall flowering plants [3]; 40% in the Canary Islands [4]) and a predominance of woody growth habit among endemics (e.g., 72% of Canary Island endemics are woody [5], many of which were derived from continental herbaceous ancestors). However, the region in general, and especially the Canary Islands, differs markedly from Pacific archipelagos such as Hawaii, Galapagos, and Juan Fernandez Islands, in that they are very close to potential continental source areas (e.g., the eastern-most Canary Island, Fuerteventura, is presently less than 100 km from the west coast of Morocco) and exhibit a comparatively old and broad range of geological ages from <1 to 21 My [2], [3]. These two features may contribute to several unusual patterns of colonization and diversification and to relatively high levels of genetic variation compared to other oceanic archipelagos [6].

The preponderance of endemic plant species has made the Macaronesian islands an outstanding area for studies of evolution and speciation, and plants from these islands have been the focus of intensive investigation in the last 15 years [7]–[10]. Numerous molecular phylogenetic analyses of Macaronesian plant groups have provided valuable insights into the relationships among the region's endemics [11]–[20]. These studies have revealed several emerging general patterns of colonization and dispersal, including: (1) single colonization of a common ancestor followed by rapid radiation [11]–[15], [18]; (2) multiple independent colonizations [16], [17], [19], [20]; (3) back-colonization to the continent from Macaronesia [15], [19], [20]; and (4) a predominantly Macaronesian-Western Mediterranean source area for the endemic lineages (see examples in [19]). Despite numerous molecular phylogenetic studies, there has been no comprehensive investigation of the timing and/or tempo of Macaronesian flowering plant radiations [10]. Previous studies often used average substitution rates of isozyme data [21], [22], cpDNA RFLP data [11], or nuclear and chloroplast DNA data [12], [13] of other organisms to estimate the origin and timing of radiation of the endemic flora, or more often made no attempt to do so [14]–[20].

Here we provide a first report the timing of origin and radiation, long distance dispersal events, and rate of diversification of five of the largest and most diverse Macaronesian plant endemic. These groups span several lineages of the core eudicots (Fig. S1, Supporting Information): the woody *Sonchus* alliance (6 genera, ca. 31 species; Asterids, Euasterid II, Asterales, [13], Fig. S2), *Echium* (27 species; Asterids, Euasterid I, unplaced, [12], Fig. S3), *Sideritis* (subgenus *Marrubiastrum*, 23 species; Asterids, Euasterid I, Lamiales, [14], Fig. S4), *Crambe* (section *Dendrocrambe*, 14 species; Rosids, Eurosid II, Brassicales, [18], Fig. S5), the *Aeonium* alliance (4 genera, ca. 61 species; Core Eudicots, Saxifragales, [15], Fig. S6). These five plant groups are premier examples of adaptive radiation in Macaronesia, each of which underwent rapid radiation after a single colonization event from continental source area, and distributed in more than one archipelago. Lack of absolute age and diversification rate estimates in such groups precludes comparisons with other insular radiations, particularly Pacific island [23, *references therein*], and continental radiations [24], [25, *references therein*], which may exhibit different patterns of diversification. In this paper we estimate the age and diversification rate of five of the largest plant lineages endemic to the Macaronesian islands. Comparison of these data for the island endemics versus their congeners on the continent is beyond the scope of this paper due to a lack of available robust phylogenetic frameworks (with exceptions in *Sonchus* and *Crambe*) and a reliable data for clock calibration.

## Results and Discussion

**Figure 2:**
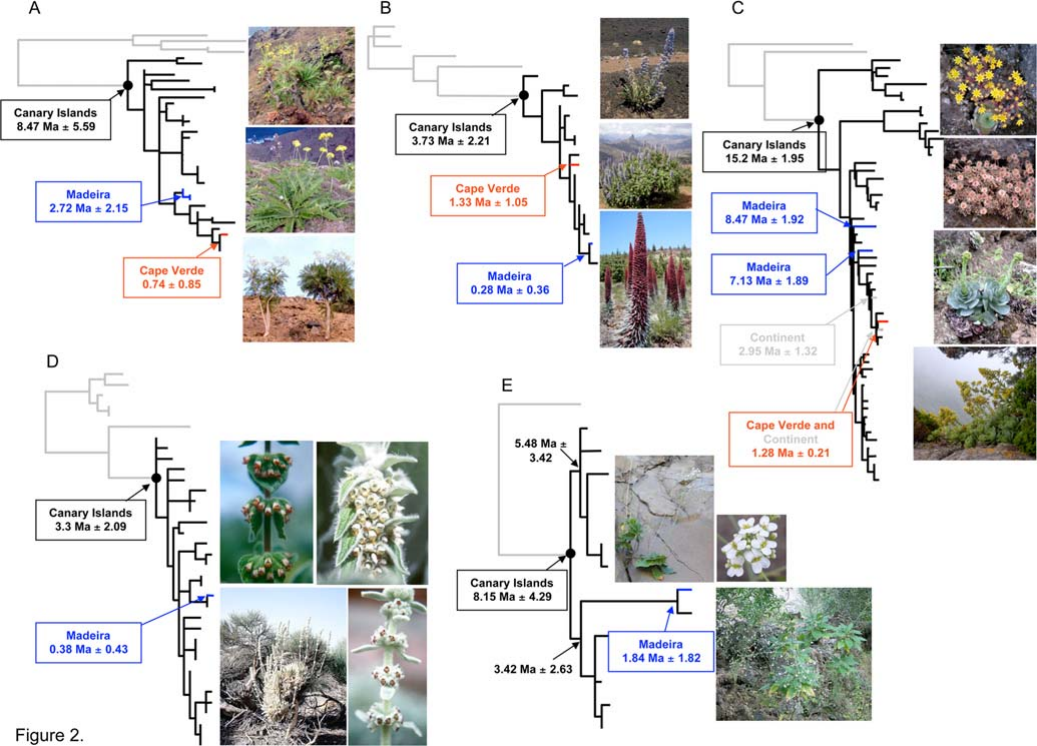
Phylogeny and age estimate of five flowering plant groups in the Macaronesian Islands. (A) the woody *Sonchus* alliance (Asterids, Euasterid II, Asterales) (species in the pictures, from top to bottom; Sonchus gandogeri*, S. acaulis*, and S. canariensis*) . (B) *Echium* (Asterids, Euasterid I, unplaced) (species in the pictures; *Echium auberianum* ^†^, E. callithyrsum*, and *E. wildpretii* ^†^). (C) the *Aeonium* alliance (Saxifragales) (species in the pictures; *Aichryson punctatum* ^∏^, *Monanthes muralis* ^∏^, Greenovia aurea*, and *Aeonium cuneatum* ^∏^). (D) *Sideritis* (Asterids, Euasterid I, Lamiales) (species in the pictures, top left, *Sideritis gomerae* ^¶^; top right, *S. macrostachys* ^¶^; bottom row, *S. eriocephala* ^¶^). (E) *Crambe* (Rosids, Eurosid II, Brassicales) (species in the pictures; *Crambe scaberrima* ^§^ and *C. pritzelii* ^§^). Branch colors: gray, closest continental relatives; black, the Canary Islands; blue, Madeira; red, Cape Verde. Branch lengths are proportional to changes on the trees and outgroup taxa are not shown. (photo credits: *Seung-Chul Kim, ^†^Jose Mesa, ^¶^Janet C. Barber, ^§^Manuel Luis Gil González, and ^∏^Mark Mort).

Three narrow windows of colonization were found for the five plant groups: Middle Miocene (early Serravllian), Late Miocene (late Tortonian), and Early Pliocene (early Zanclean). The *Aeonium* alliance, a well supported clade of three genera, showed the earliest colonization into the Canary Islands (15.2 Ma), followed by the woody *Sonchus* alliance (8.47 Ma), *Crambe* (8.15 Ma), *Echium* (3.73 Ma), and *Sideritis* (3.3 Ma) (Fig. 2 and Table 1). The most recent common ancestor (MRCA) of the *Aeonium* alliance colonized the Canaries when the two eastern-most, geologically older islands were formed during early to mid Tertiary. Within the *Aeonium* alliance, the MRCA ages of *Aichryson*, *Monanthes*, and *Aeonium* (including *Greenovia*) are estimated to be 8.67 Ma, 6.93 Ma, and 10.23 Ma, respectively. These divergence time estimates suggest that after the initial colonization, three major lineages slowly diverged and radiation of each lineage began during late Miocene. A second episode of synchronized colonization during the Late Miocene was inferred for Eurosid II and Euastrid II lineages: *Crambe* (Brassicaceae) and the *Sonchus* alliance (Asteraceae). Lastly, two of the most speciose and recently radiated lineages among the five investigated here are both members of the families in Euasterid I clade: *Sideritis* (Lamiaceae) and *Echium* (Boraginaceae); we infer that these taxa colonized the Canaries during the early Pliocene.

**Table 1:** Absolute age estimates of MRCA (most recent common ancestor) in the Macaronesian Islands, colonization events into other archipelagos (i.e., Madeira and Cape Verde), and back dispersal to mainland Africa (21 My hard bound analyses based on the oldest age of the Canary archipelago are in brackets).

	Root Age	MRCA	Dispersal Into	Dispersal Into	Back Dispersal	Rate of Diversification
*Sonchus*	13.203±5.51	8.475±5.50	2.717±2.15	0.739±0.84	NA	0.32339 (0.21472–0.65815)
*Echium*	7.931±3.58	3.733±2.21	0.282±0.36	1.328±1.05	NA	0.6168 (0.387–1.51228)
*Aeonium* alliance	18.833±1.81	15.263±1.95	8.47±1.92, 7.13±1.89	1.281±0.92	2.95±1.32, 1.28±0.21	0.2159 (0.1914–0.2476)
*Sideritis*	11.923±5.87	3.329±2.09	0.380±0.43	NA	NA	0.7922 (0.4479–3.4262)
*Crambe*	14.885±5.52	8.158±4.29	1.839±1.82	NA	NA	0.2385 (0.1562–0.504)

The absolute age estimates by the Multidivtime represent mean values followed by standard deviations. The diversification rate based on mean value is shown followed by the ranges using the upper and lower bounds of standard deviation.

The closest continental ancestor(s) of each of the five lineages investigated is inferred to be from the western Mediterranean, including the Iberian Peninsula and Morocco and the timing of colonization coincides with major geological and climatic changes in those areas. The first colonization event by the *Aeonium* alliance occurred during the Betic crisis (16–14 Ma), which was characterized by significant fluctuations in temperature and resulting glacioeustatic changes in global sea level [26]. We hypothesized that the MRCA of the *Aeonium* alliance colonized the geologically older eastern islands first and subsequently dispersed to the later forming western islands (Gran Canaria, Tenerife, and La Gomera). Second episode of colonization is synchronous with the onset of recurrent desert conditions in the Sahara [27] and occurred prior to the desiccation of the Mediterranean Sea (i.e., Messinian salinity crisis, [28]) when seasonal contrasts in the temperature regime developed. The colonization events of these two unrelated genera were perhaps triggered by these two dramatic geological events in the Mediterranean region. At this time, all but two of the western Canary Islands were formed and it is highly likely that these two genera colonized Tenerife and Gran Canaria separately. A third episode of colonization is inferred for *Echium* and *Sideritis*, coinciding with the opening of the Gibraltar Strait and the onset of the first glaciation cycles [29]. These three discrete waves of colonization support the “Colonization Window Hypothesis” which implies that opportunity for island colonization may have been constrained to one or more distinct periods of time [30]. It is possible that three discrete time periods of profound climatic and geologic changes in northern Africa and the Mediterranean and of active volcanism in the Canary Islands facilitated the establishment and subsequent diversification of endemic lineages. The colonization of *Limonium* (Plumbaginaceae) subsection *Nobiles* (7.5 Ma, [31]) into the Canaries coincides with the second wave of colonization of *Sonchus* and *Crambe* (i.e., prior to the desiccation of the Mediterranean Sea in the Messinian). The absolute ages of the five genera pre-date those of later multiple colonizers which did not undergo adaptive radiation, such as *Convolvulus* [30], and this can be explained by the niche pre-emption hypothesis: earlier colonists that radiated created a clade or clades that occupied more niche space than later colonists that did not radiate. Thus, by filling niche space, adaptive radiation created a barrier that prevented closely related later arrivals from establishing [32].

Inter-archipelago dispersal events in Macaronesia, with an exception of the *Aeonium* alliance dispersal into Madeira, appear to be quite recent and did not result in any major radiation for any of these five genera. Our data suggest that dispersal to Madeira occurred in the Late Pliocene and Pleistocene, between 3 and 0.3 Ma, and this time-window is coincident with the onset of the first glaciation cycle. Dispersal into the Cape Verde Islands of three plant groups is even more recent, i.e., during Pleistocene. Back dispersal of *Aeonium* to the mainland African continent also occurred between 3.0 and 1.28 Ma without subsequent major speciation or radiation. The five lineages investigated here illustrate a variety of dispersal syndromes (*Aeonium* alliance and *Crambe*, unassisted; *Sonchus* alliance, hydrochory; *Echium* and *Sideritis*, epizoochory: [10]). It is plausible that different dispersal mechanisms facilitated inter-archipelago colonization during glaciation cycles.

The estimated mean per lineage diversification rate per million years ranges from 0.22 to 0.79 (Table 1 and Fig. 2). The earliest inferred colonizer, the *Aeonium* alliance, appears to have the slowest rate of diversification (0.22 for the entire alliance; *Aichryson* only, 0.13; *Monanthes* only, 0.20; *Aeonium* including *Greenovia* only, 0.30), whereas the most recent colonizing lineages (i.e., *Echium* and *Sideritis*) have the fastest diversification rates (0.62 and 0.79, respectively). The diversification rates of *Aeonium* alliance and *Crambe* (0.24) are on the low end of the range inferred for other continental and insular radiations (e.g., angiosperm families [33], rodent families [34], and recent African large-mammal genera [35], [36]). We estimated the rate of diversification for the *Sonchus* alliance to 0.32 (maximum of 0.66), which is lower than the rate of the silversword alliance in Hawaii (0.56), but similar to the rate of *Agave* sensu lato (0.32) [37]. The rates of diversification in the later colonizers, *Echium* and *Sideritis* lineages, were about two to three times greater than those of earlier colonizers. These rates exceed that of the Hawaiian silversword alliance [23] and are comparable to Neogene horses (0.5–1.4) [38], Lake Tanganyika cichlids (0.75–1.49) [39], Southern African semi-desert ice plants (0.77–1.75) [24], and Angiosperm orders (maximum of 0.76) [40]. These results suggest that, unlike some other radiations, the Macaronesian island endemics show moderate to relatively rapid speciation rates depending on plant groups. Common possible mechanisms driving diversification are difficult to postulate. During the late Miocene and Pleistocene, however, it is likely that multiple catastrophic volcanic episodes, increasing altitudinal gradients, and the onset of moisture-laden northeastern trade winds created a remarkable array of ecological habitats. In addition, several other major geological and climatic changes in western Mediterranean areas including the Iberian Peninsula, northern Africa, and western Europe could possibly have promoted rapid speciation, inter-island and inter-archipelago colonization of those plant groups.

In conclusion, we recover three discrete windows of colonization for the Canary Islands that explain the origin and adaptive radiation of five major eudicot lineages in Macaronesia. Inter-archipelago dispersals into Madeira and the Cape Verdes appear to be quite recent without major subsequent diversification in these archipelagos. The tempo of adaptive radiation of major Macaronesian plant endemics varies from moderate to relatively rapid speciation rates.

## Materials and Methods

### Selection of plant groups

The flora of the Macaronesian Islands [1], which encompasses five Atlantic archipelagos, is exceptionally rich and diverse. The number of flowering plant species is approximately 3200, of which some 680, or 20%, are endemic [1], [2], [41]. Among the five archipelagos, the Canary Islands (percentage of endemics, 25.5% of the whole flora and 40% of the native flora) are by far the richest and most diverse group of islands followed by the Cape Verde (15%), Madeira (8%), the Azores (5.2%), and the Salvage Islands (2.2%) [3]. Three archipelagos, Madeira, The Canaries, and the Azores, share most of these taxa [1], [3] and the Cape Verde group are shared largely by the Canaries [1], [42]. The Salvage Islands (area of <15 km^2^ with highest altitude of <200 m) are intermediate in their floristic character between the Canary Islands and Madeira. Selection of plant groups for this study was based on three criteria: (1) availability of molecular phylogenetic study based on DNA sequence, (2) phylogenetic position in the Angiosperm classification system, and (3) monophyletic assemblage of groups in the Macaronesian islands. Based on these criteria, we selected five core eudicot lineages that were previously shown to be monophyletic in Macaronesia (Supporting Information, Figure S1). Monocot representatives were not included since none of them represent diverse monophyletic groups in Macaronesia. The largest endemic plant genus found on volcanic islands in the Atlantic Ocean, *Argyranthemum* (Asteraceae), was also excluded due to lack of available DNA sequence data; previous studies used chloroplast DNA restriction-site variations and allozyme frequency data to infer the evolutionary history of the group [43]. In addition, several other plant groups with available molecular phylogenetic studies based on DNA sequence were excluded because they represent multiple colonizations into Macaronesia, are comparatively small plant group, are restricted to one archipelago, and/or they are considered closely related to groups already sampled (e.g., *Saxifraga* [44], *Bencomia* Alliance [45], *Gonosperminae* [46], *Euphorbia* [47], *Asteriscus* [48], *Lavatera* [17], *Broom* species [16], *Convolvulus* [19], *Lotus* [20], *Bystropogon* [49], *Limonium* [31]).

The selection of five plant groups, thus, best represents the most diverse monophyletic plant groups in Macaronesia in which adaptive radiation is a primary explanation for morphological and ecological diversification. All five lineages display predominantly woody life forms in Macaronesia and are considered Tertiary relicts and ancestral to modern Mediterranean relatives [50]. They are distributed in more than one archipelago, primarily in the Canaries, Madeira, and the Cape Verde Islands. Recent molecular phylogenetic studies [12]–[15], [19] demonstrated that the initial colonization and diversification occurred recently on the Canary archipelago from the western Africa/Iberian Peninsula and Mediterranean region, and refuted the relictual nature of the woody-life forms.

### Sequence data and phylogenetic analysis

For the woody *Sonchus* alliance (Asteraceae, subgenus *Dendrosonchus* and five allied genera), the data matrix based on the internal transcribed spacer (ITS) of nuclear ribosomal DNA (nrDNA) [51] was used. Approximately 4000 base pairs (bp) of chloroplast DNA (cpDNA) coding and noncoding sequences were highly invariable and the combined data analysis showed almost identical tree topology as the one based on ITS only [13]. Therefore, we used the ITS data matrix only to estimate the absolute age and diversification rate. The closest continental relatives of the alliance turned out to be *Sonchus* subgenus *Sonchus* the Ibero-African section *Pustulati* [52], and thus we used a representative of this section, *Sonchus palustris*, which occurs widely in western non-Mediterranean region, as an outgroup. The data matrix included three noncoding cpDNA gene regions and nrDNA ITS 1 sequences [53] was used for genus *Echium* (Boraginaceae). Five continental species were used as outgroups based on the previous study [12] and five additional species, primarily Iberian and Mediterranean, were used as closest continental species for the Macaronesian *Echium* species. For the ITS data matrix of genus *Crambe* (Brassicaceae, subgenus *Dendrocrambe*), two species *C. kilimandscharica* and *C. orientalis*, were used as outgroups [18], while *C. kralikii* which occurs in Morocco, was used as sole continental closest relative. For *Sideritis* (Lamiaceae, subgenus *Marrubiastrum*), four widespread annual and eastern Mediterranean perennial species were used as outgroups, while five primarily western Mediterranean perennials and one annual species from Morocco were used as closest continental species. The ITS data matrix [14] was used for this genus since ITS tree was better resolved, more strongly supported, and reflected the relationships based on morphology better than the cpDNA based tree. Lastly, for the *Aeonium* alliance (*Aeonium*, *Aichryson*, *Monanthes*, and *Greenovia*), we used the same ITS and cpDNA (*psb*A-*trn*H and *trn*L-*trn*F) data set [15]; however, *mat*K sequences were excluded because they were not highly variable. Two species, *Sedum clavatum* and *Echeveria fulgens*, in the *Acre* clade, which is sister to *Aeonium* [54] were used as outgroups, while two other species of *Sedum*, *S. jaccardianum* and *S. modestum*, which occur in northern Africa, were used as closest continental relatives. All five of the Macaronesian lineages included in this study have complete or nearly complete species sampling. Life history effects on ITS substitution rate is likely negligible since all five plant groups have similar life forms, i.e., long-lived woody perennials.

Maximum likelihood (ML) analysis was performed for each group (see Supporting Information, Figures S2, S3, S4, S5 and S6). The best fit model based on the Akaike Information Criterion (AIC) implemented in ModelTest [55] was chosen. Model parameters were then imported into PAUP* [56], and heuristic searches were executed. ML bootstrap analyses with 100 replicates were conducted using the same parameter values obtained from ModelTest.

### Divergence time estimates

Maximum likelihood trees (as described above) of each of the five Macaronesian clades were used to estimate divergence dates using Bayesian methods implemented in Multidivtime [57], [58]. ML parameters were estimated for each tree using the F84+G model in PAUP*. The estbranches package was employed to estimate branch lengths and generate a variance-covariance matrix using these parameters. Multidivtime was then used to produce a posterior probability distribution to calculate the mean and standard deviation of divergence times using default settings for burn-in length and sampling frequency of the Markov chain [58].

### Molecular dating calibration

Unfortunately, paleobotanical and paleoclimatic data do not provide irrefutable evidence for the ages of the Macaronesian flora. Terrestrial plant fossils dated at 13 My before present (BP) have been recorded from Gran Canaria, and fossils of several plant taxa that are currently restricted to or have distributions centered on Macaronesia have been discovered in continental Europe [1], [59], [60]; however, the precise taxonomic identification of these fossils has proved inconclusive [61].

Given the lack of reliable fossil and/or paleoclimatic data, we used well-documented geological estimates of island ages as our calibration points. The oldest Macaronesian island, Fuerteventura, is a member of the Canary Islands, and is estimated to be approximately 21 million years old (Early Miocene). Older seamounts surrounding Macaronesia have been dated to 68 myr [62], but these are much too old to date divergences within plan families that originated in the Tertiary: Asteraceae, mid Oligocene, ca. 30 Myr [24], [63]; Brassicaceae, Oligocene, ca. 30 Myr 64, 65; Lamiales s.l., late Eocene/Oligocene, ca. 35 Myr [66], [67]; Boraginaceae, subfamily Boraginoideae, Oligocene, ca 32 Myr [68]; Crassulaceae, mid Eocene, ca. 40 Myr, split between *Sedum*/*Dudleya* and *Kalenchoe*, <30 Myr [67]. Therefore, 21 My serves as a conservative maximum age for the most recent common ancestor of the clade comprising the Macaronesian endemics and their continenetal relatives. The upper limit of 21 My is within the range of the Miocene and Pliocene fossil flora in southern Europe that are present today in Macaronesia [1], [50] and some of the dramatic paleogeological/paleoclimatic events in northern Africa and Europe (e.g., the Betic crisis [15–16 mya], the Messinian salinity crisis [6.5-5.5 mya], [69]–[71], and the Sahara desertification [7 mya], [72]). Both purported ancestral islands of each group (Tenerife and Gran Canaria, 12 and 14 Ma, respectively) and the oldest Macaronesian islands (Fuerteventura, 21 Ma) were used as root calibration points; the date of the oldest island (21 Ma) was used as a hard upper bound at the root of each tree.

### Species diversification rate

Species diversification rates were estimated using a simple pure-birth macroevolutionary model given by the formula *D = (ln N_i_−ln N_0_)/T*, where *N_i_* equals the number of extant species and *N_0_* equals the number of species at time *T*, the estimated age of the most recent common ancestor in millions of years.

## Supporting Information

Figure S1 Phylogenetic classification of the flowering plants and the position of five plant groups studied. The tree is from the Angiosperm Phylogeny Group (version 7, June 2007) (http://www.mobot.org/MOBOT/Research/APweb/).
(3.06 MB TIF)

Figure S2 Maximum likelihood phylogram (model: SYM+G, -ln = 1579.7311) of the woody Sonchus alliance (Asteraceae) based on ITS sequence of nrDNA. Gray circle represents calibration point. (S. = *Sonchus*) (1 = dispersal to the Canary Islands, 2 = dispersal to Madeira, 3 = dispersal to Cape Verde).
(0.96 MB TIF)

Figure S3 Maximum likelihood phylogram (model: GTR+I+G, -ln = 3228.8472) of *Echium* (Boraginaceae) based on ITS and cpDNA sequences. Gray circle represents calibration point. (1 = dispersal to the Canary Islands, 2 = dispersal to Cape Verde, 3 = dispersal to Madeira).
(0.79 MB TIF)

Figure S4 Maximum likelihood phylogram (model: TIM+G, -ln = 2110.9453) of *Sideritis* (Lamiaceae) based on ITS sequence. Gray circle represent calibration point. (1 = dispersal to the Canary Islands, 2 = dispersal to Medeira).
(0.89 MB TIF)

Figure S5 Maximum likelihood phylogram (model: K80+G, -ln = 1176.8682) of *Crambe* section *Dendrocrambe* (Brassicaceae) based on ITS sequence. Gray circle represents calibration point. (1 = dispersal to the Canary Islands, 2 = dispersal to Madeira).
(0.83 MB TIF)

Figure S6 Maximum likelihood phylogram (model: TIM+I+G, -ln = 12034.2559) of the *Aeonium* alliance (Crassulaceae) based on ITS and cpDNA sequences. Gray circle represents calibration point. (1 = Canary Islands, 2 and 2′ = dispersal to Madeira, 3 and 3′ = dispersal to continent, 4 = dispersal to Cape Verde).
(0.93 MB TIF)
