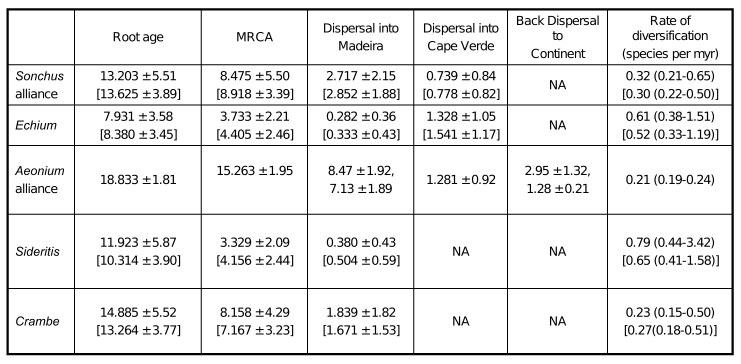# Correction: Timing and Tempo of Early and Successive Adaptive Radiations in Macaronesia

**DOI:** 10.1371/annotation/a8922076-0da4-41ff-94ef-5e44f60d1895

**Published:** 2008-05-20

**Authors:** Seung-Chul Kim, Michael R. McGowen, Pesach Lubinsky, Janet C. Barber, Mark E. Mort, Arnoldo Santos-Guerra

There were formatting errors and missing data in Table 1. The corrected table is available here:

**Figure:**