# Supplementary figures and images for: Timing and Tempo of Early and Successive Adaptive Radiations in Macaronesia
Source: PLoS One. 2008 May 14;3(5):e2139. doi: 10.1371/journal.pone.0002139 (PMC2367450; doi:10.1371/journal.pone.0002139)

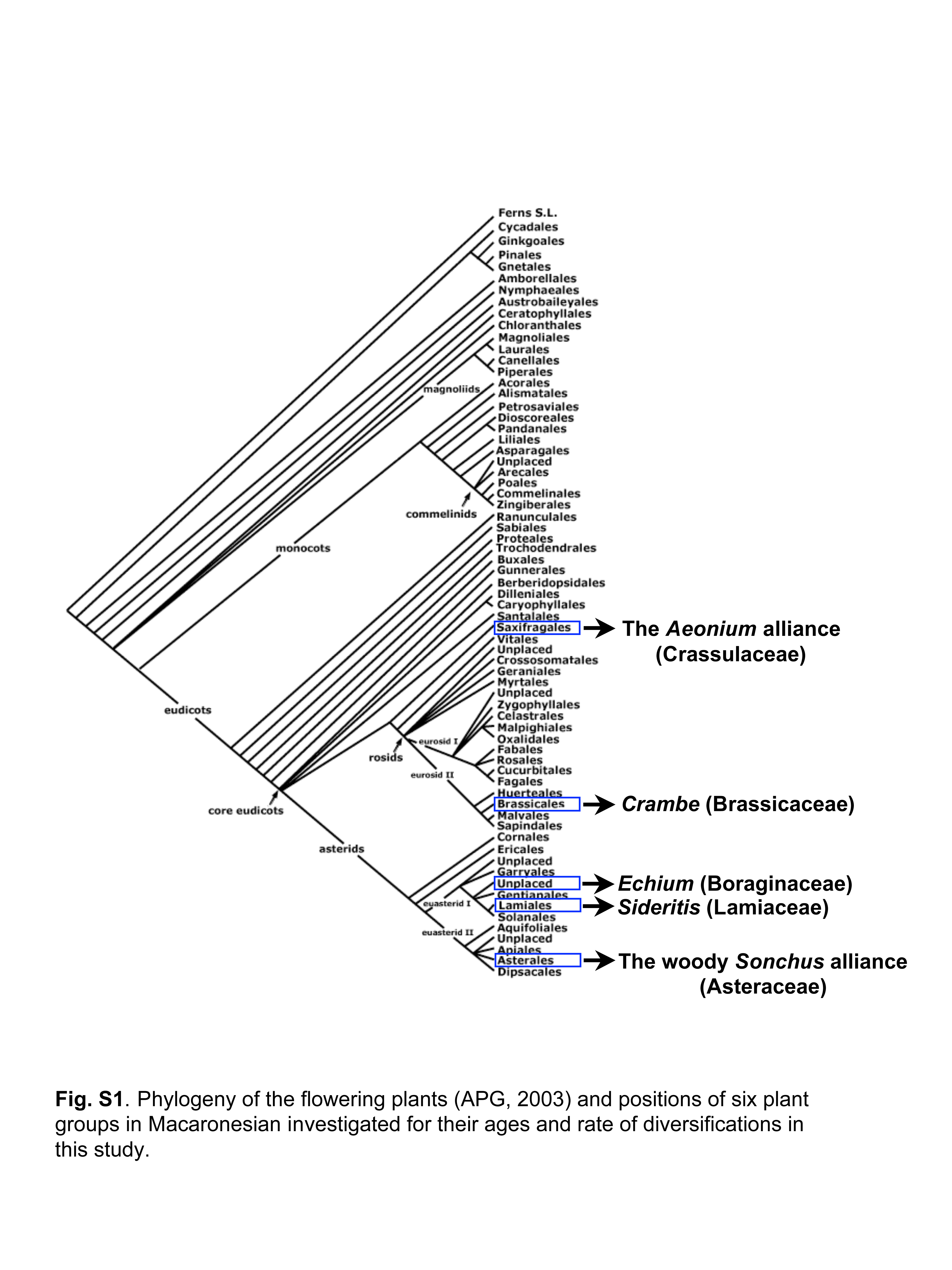

Supplement: Figure S1 — Phylogenetic classification of the flowering plants and the position of five plant groups studied. The tree is from the Angiosperm Phylogeny Group (version 7, June 2007) (http://www.mobot.org/MOBOT/Research/APweb/). (3.06 MB TIF) [file pone.0002139.s001.tif]

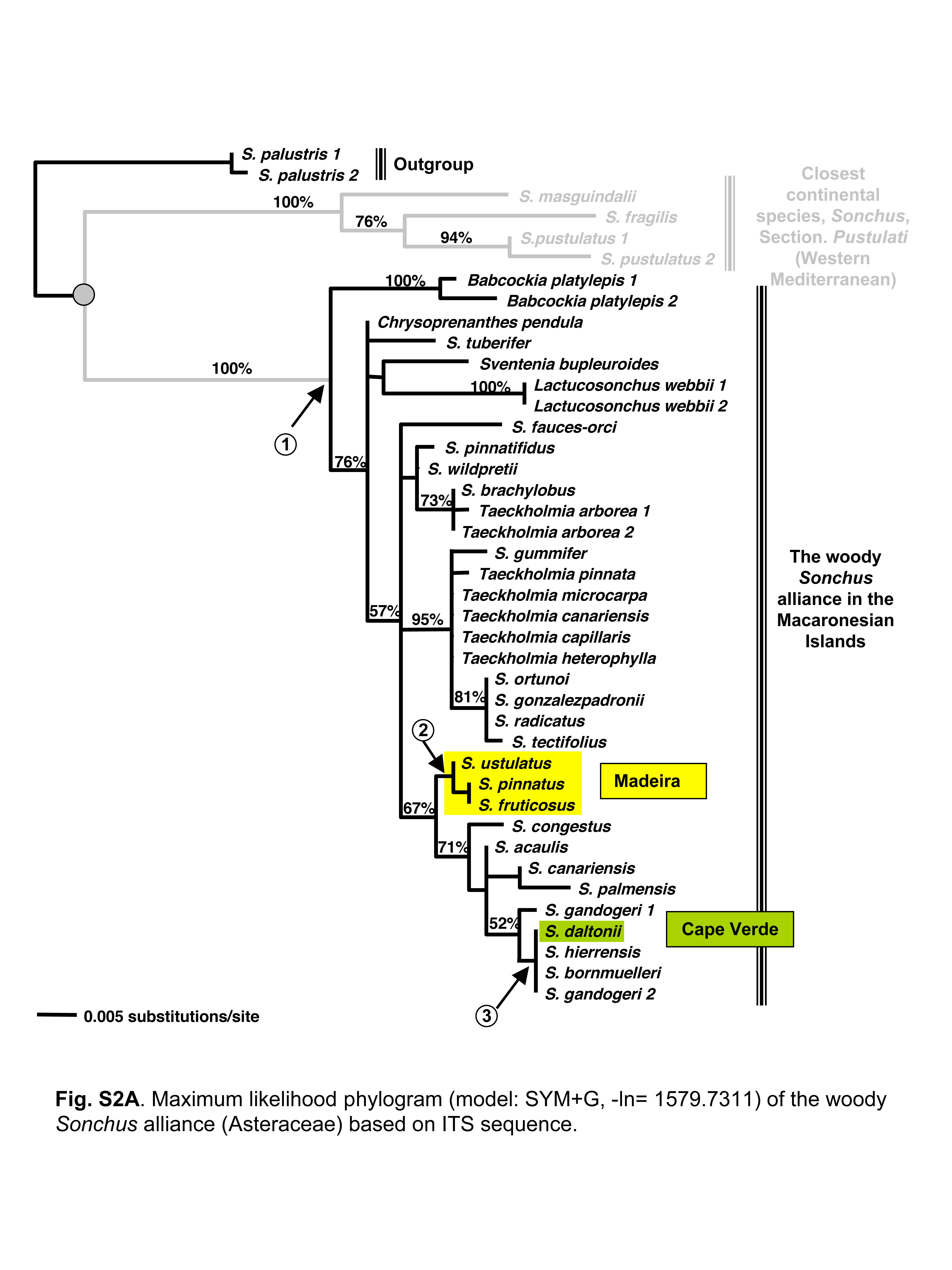

Supplement: Figure S2 — Maximum likelihood phylogram (model: SYM+G, -ln = 1579.7311) of the woody Sonchus alliance (Asteraceae) based on ITS sequence of nrDNA. Gray circle represents calibration point. (S. = Sonchus) (1 = dispersal to the Canary Islands, 2 = dispersal to Madeira, 3 = dispersal to Cape Verde). (0.96 MB TIF) [file pone.0002139.s002.tif]

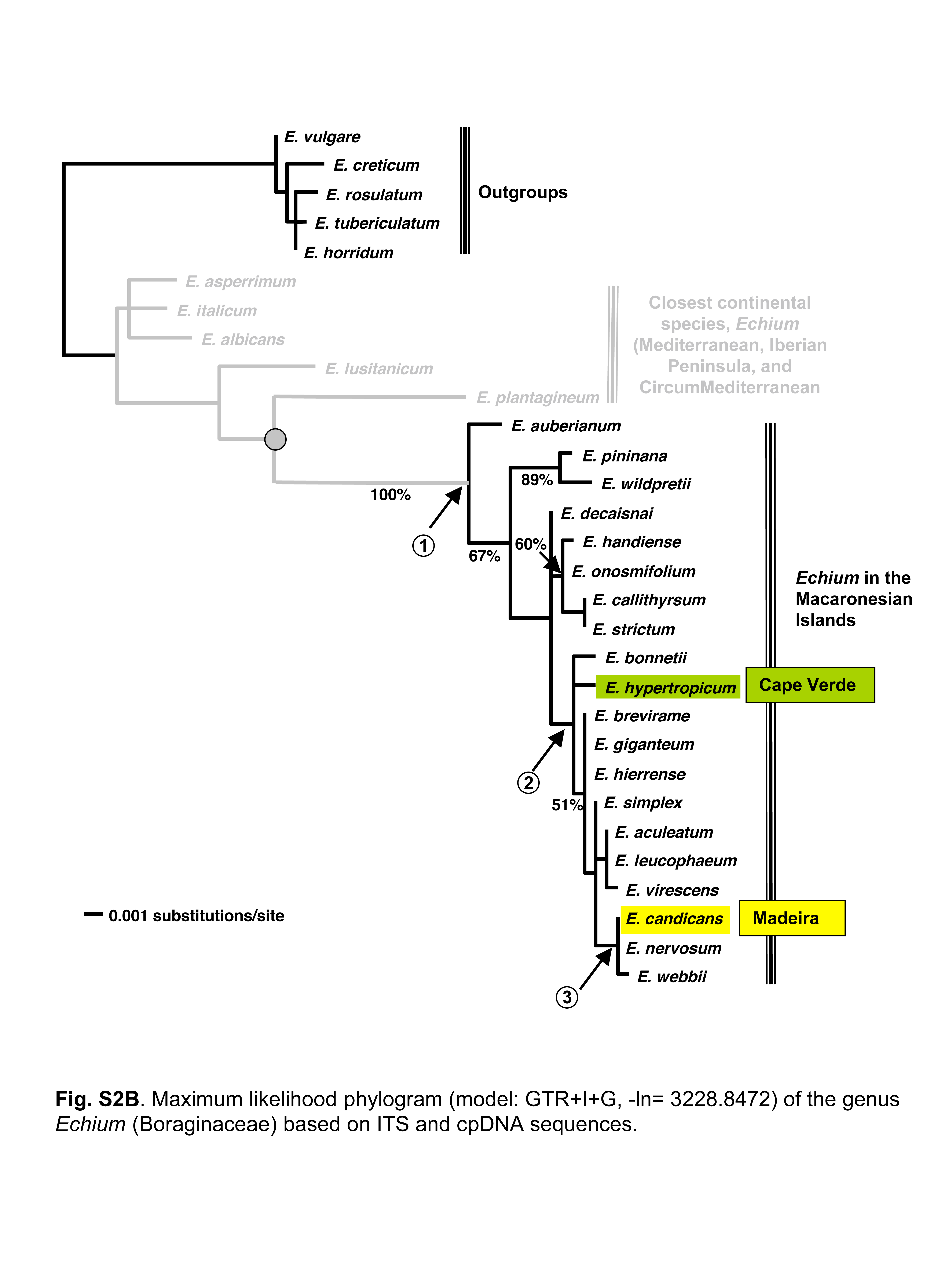

Supplement: Figure S3 — Maximum likelihood phylogram (model: GTR+I+G, -ln = 3228.8472) of Echium (Boraginaceae) based on ITS and cpDNA sequences. Gray circle represents calibration point. (1 = dispersal to the Canary Islands, 2 = dispersal to Cape Verde, 3 = dispersal to Madeira). (0.79 MB TIF) [file pone.0002139.s003.tif]

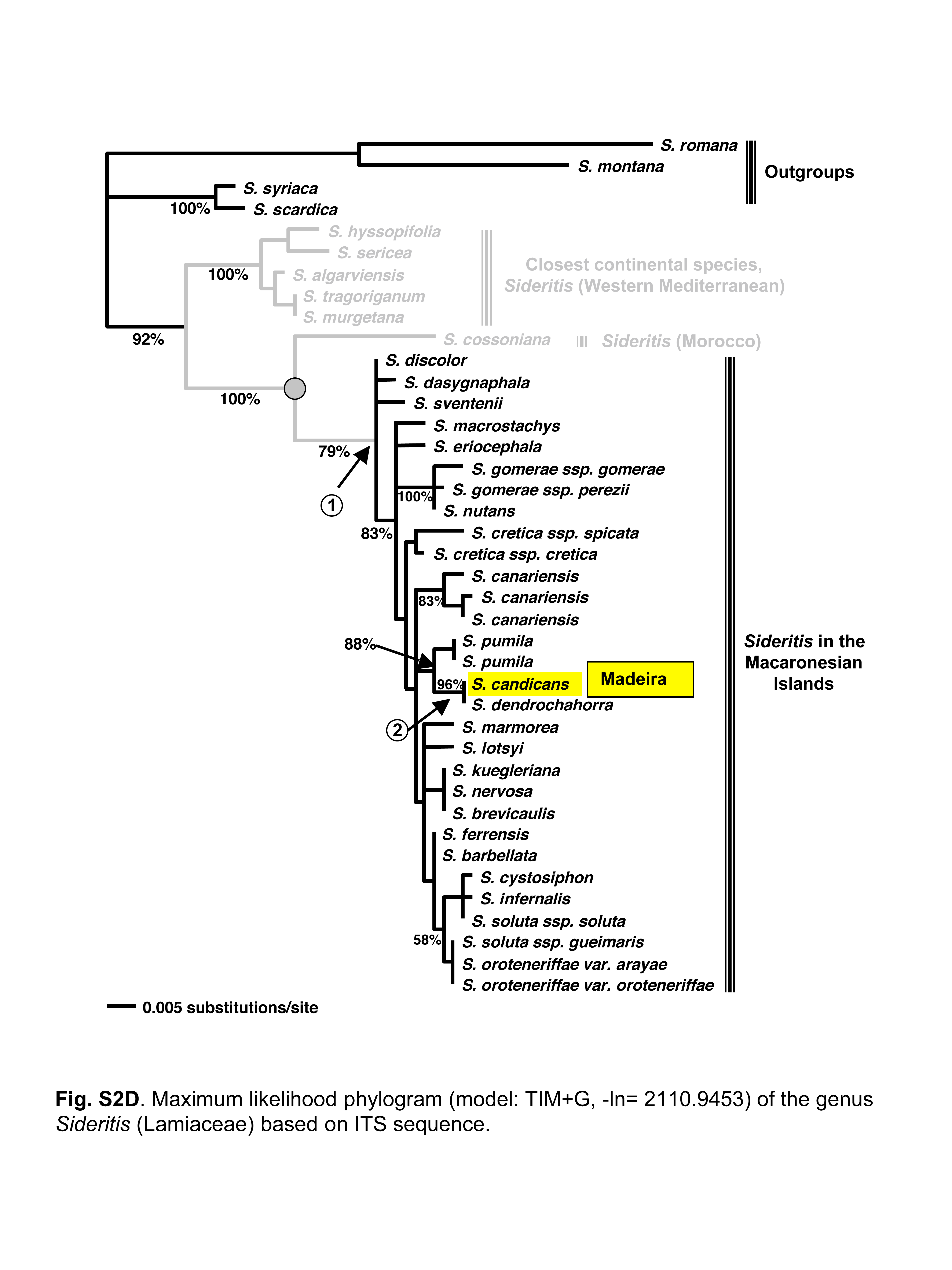

Supplement: Figure S4 — Maximum likelihood phylogram (model: TIM+G, -ln = 2110.9453) of Sideritis (Lamiaceae) based on ITS sequence. Gray circle represent calibration point. (1 = dispersal to the Canary Islands, 2 = dispersal to Medeira). (0.89 MB TIF) [file pone.0002139.s004.tif]

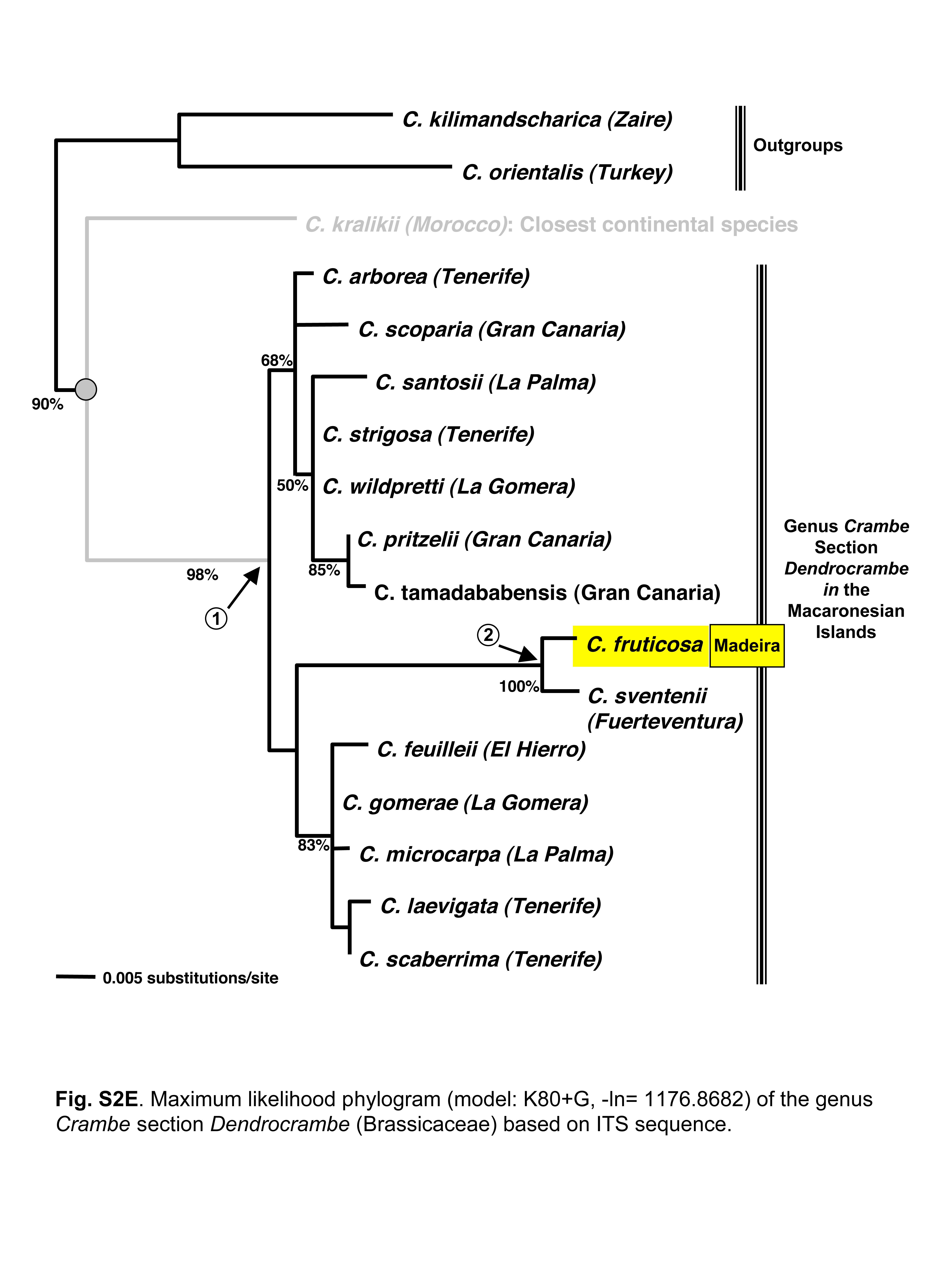

Supplement: Figure S5 — Maximum likelihood phylogram (model: K80+G, -ln = 1176.8682) of Crambe section Dendrocrambe (Brassicaceae) based on ITS sequence. Gray circle represents calibration point. (1 = dispersal to the Canary Islands, 2 = dispersal to Madeira). (0.83 MB TIF) [file pone.0002139.s005.tif]

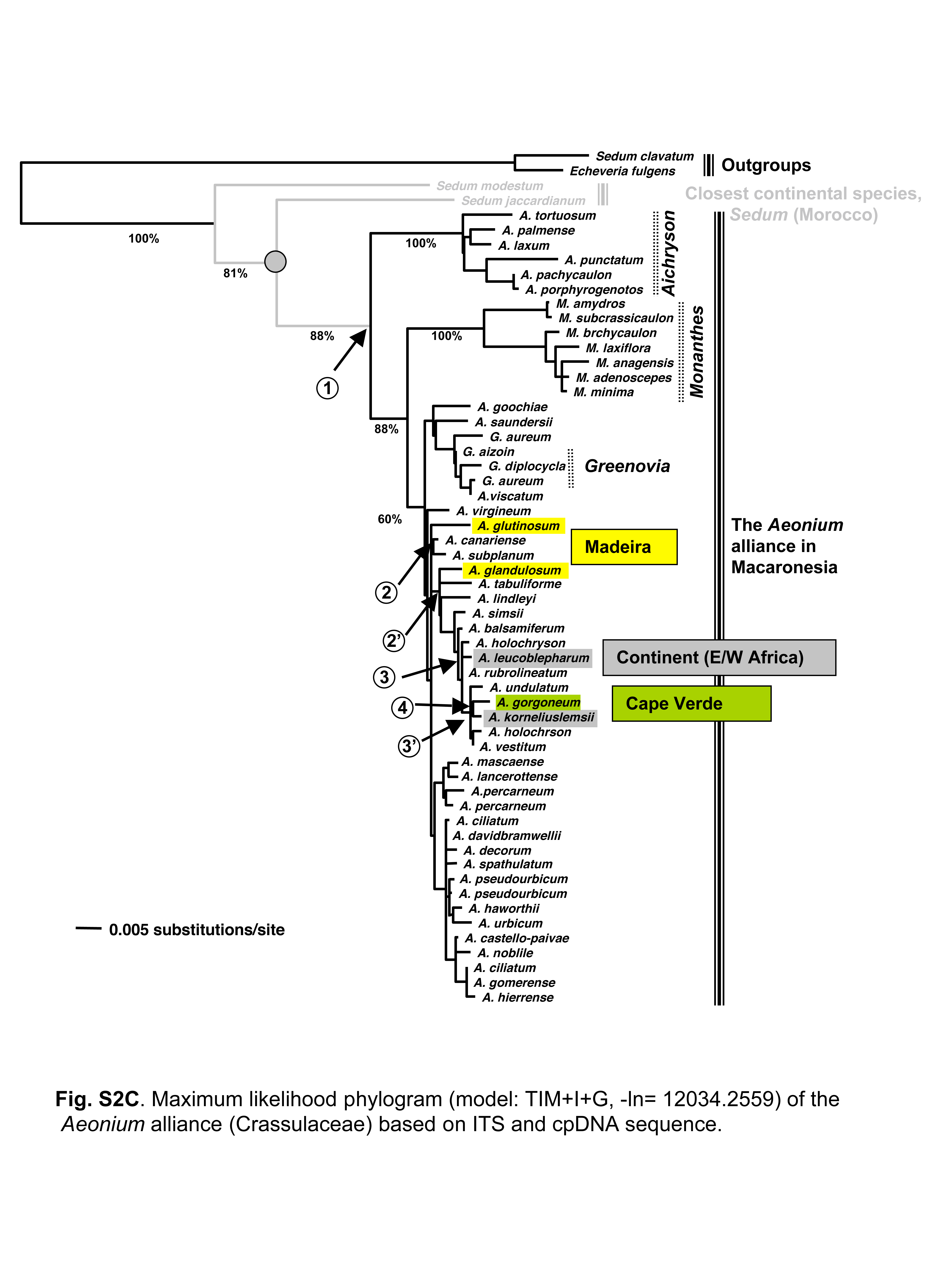

Supplement: Figure S6 — Maximum likelihood phylogram (model: TIM+I+G, -ln = 12034.2559) of the Aeonium alliance (Crassulaceae) based on ITS and cpDNA sequences. Gray circle represents calibration point. (1 = Canary Islands, 2 and 2′ = dispersal to Madeira, 3 and 3′ = dispersal to continent, 4 = dispersal to Cape Verde). (0.93 MB TIF) [file pone.0002139.s006.tif]
